# Oral tribology of dairy protein-rich emulsions and emulsion-filled gels affected by colloidal processing and composition

**DOI:** 10.1016/j.crfs.2024.100806

**Published:** 2024-07-14

**Authors:** Andrea Araiza-Calahorra, Alan R. Mackie, Anwesha Sarkar

**Affiliations:** Food Colloids and Bioprocessing Group, School of Food Science and Nutrition, University of Leeds, Leeds, LS2 9JT, UK

**Keywords:** Lubrication, Bolus, Whey protein hydrolysate, Viscosity, Oral processing

## Abstract

Designing nutritious food for the elderly population often requires significant quantities of leucine-rich whey proteins to combat malnutrition, yet high-protein formulations can cause mouth dryness and increased oral friction. This study investigated how various colloidal processing methods and compositions impact the *in vitro* oral tribological properties of protein-rich emulsions and emulsion-filled gels. Oil-in-water emulsions with oil fractions from 1 wt% to 20 wt% were prepared, alongside emulsion-filled gels containing whey protein isolate (WPI), hydrolysed whey protein (HWP), or a blend of both (10 wt% protein content). Two processing approaches were employed: creating emulsions with an initial 10 w% protein content (M1) and initially forming emulsions with 0.1 wt% protein content, then enriching to a final 10 wt% concentration (M2). The hypothesis was that formulations with HWP or method 2 (M2) would offer lubrication benefits by inducing droplet coalescence, aiding in the formation of a lubricating boundary tribofilm. Surprisingly, the tribological behavior of high-protein emulsions showed minimal dependence on oil droplet volume fraction. However, both HWP-based emulsions and those processed with M2 for WPI exhibited significant friction reduction, which may be attributed to the presence of coalesced oil droplets, supporting our hypothesis. Substituting 50 wt% of WPI with HWP in emulsion-filled gel boli resulted in very low friction coefficients in the boundary lubrication regime, suggesting oil droplet release from the gel matrix. These findings provide insights into designing high-protein foods with improved mouthfeel for the elderly population, necessitating further validation through sensory studies.

## Highlights

• High protein emulsions suffer from poor lubricity despite 20 wt% oil content.
• Hydrolysed whey proteins as emulsifiers make droplets prone to coalescence.
• Such hydrolysed protein stabilised emulsions improve lubricity.
• Emulsion gel boli provide limited lubricity unless hydrolysed whey protein is used.
• Model saliva tend to affect lubricity of less stable emulsions and emulsion gels.

## Graphical abstract

**Figure:**
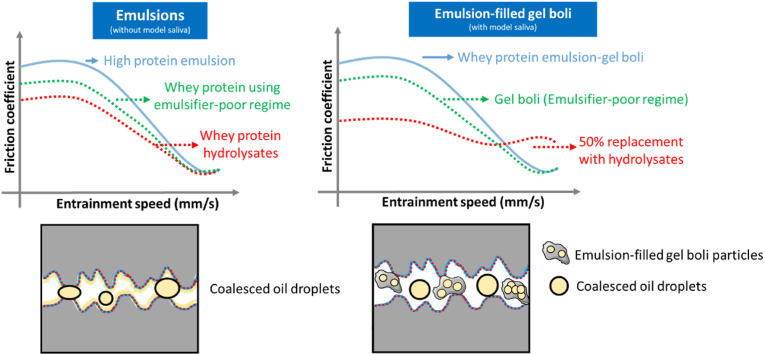

## 1 Introduction

Nutrient-dense foods ensure the delivery of essential vitamins and minerals, which is particularly important for vulnerable populations, such as the aging population (Dardevet et al., 2021). In this specific demographic, that face multiple ageing-related decline in eating capabilities, as well as health challenges such as hypertension, cardiovascular diseases, osteoporosis and sarcopenia, the development of food products that address their specific dietary requirements has become a vital research area for the food industries in recent years (Aguilera and Covacevich, 2023; Araiza-Calahorra et al., 2023; Calligaris et al., 2022; Laguna et al., 2015; Sarkar, 2019). For example, nutritional habits that follow a low-fat, low-sodium, and high-protein regime have been extensively acknowledged for their significant potential in effectively mitigating these health challenges. Currently, the most promising nutritional approach for delaying muscle loss, sarcopenia, and consequently preventing early loss of independent living and several comorbidities, involves a tailored nutritional plan that emphasizes intake of whey proteins, containing leucine, a well-documented muscle protein synthesis stimulator (Naseeb and Volpe, 2017; Rennie, 2009; Volpi et al., 2003).

Nevertheless, while the inclusion of whey protein offers several formulation benefits, using elevated concentrations to create high-protein products can introduce several mouthfeel-related disadvantages. In particular, fortifying food products with whey proteins results in dry sensation in the mouth often linked to protein-saliva interactions, that intensifies with age, potentially limiting both food consumption and overall satisfaction (Beecher et al., 2008; Withers et al., 2014). Thus, protein-fortified products commonly encounter low consumer acceptance due to associations with unfavourable taste and mouthfeel characteristics such as mouth dryness, chalkiness, metallic sensations, and a lingering film-like texture (Bull et al., 2017, Carunchia Whetstine et al., 2005, Wright J.W.Carunchia-Whetstine M.E.Miracle R.E.Drake, 2006).

Oral friction is often considered as a relevant quantitative method to provide physical understanding behind sensory dryness, astringency and lubrication (Giles et al., 2024; Lesme et al., 2024; Sarkar and Krop, 2019; Vlădescu et al., 2023). To date, only few studies have focused on the exploration of the lubrication attributes of model liquid emulsion or semi-solid protein-rich emulsion gel-based food matrices (Campbell, Foegeding and van de Velde, 2017; Chojnicka, Sala, de Kruif and van de Velde, 2009; Devezeaux de Lavergne et al., 2016; Fuhrmann et al., 2020a, Fuhrmann et al., 2020b;K. Liu, M. Stieger, E. van der Linden, & F. van de Velde, 2015; Shahbazi et al., 2021; Yang et al., 2020). Many of these studies have established empirical correlations between sensory attributes evaluated by a sensory panel and corresponding rheological and frictional measurements. Notably, these investigations have unveiled that the lubrication properties, as depicted by the pattern of the Stribeck curves, are significantly influenced by factors such as oil droplet volume fraction, interactions between emulsion droplets and matrix and the ease of fat droplet release (*e.g.* droplets acting as *inactive* fillers), and the type of dairy protein employed.

There is in fact a growing consensus that, when a fat/oil is present, a matrix that facilitates release of oil plays a central driving mechanism for stronger fat-related perceptions, attributed to the generation of a boundary oil-based film enhancing lubrication performance (Liu, Stieger, van der Linden and van de Velde, 2015). For an in-depth discussion on oral processing and the application of tribology in studying food oral processing, readers are referred to the works of Chen and Stokes (2012), Sarkar et al. (2019), Selway and Stokes (2014), and Shewan et al. (2020). This topic is considered beyond the scope of this article but is comprehensively covered in the cited reviews.

Although tribology of whey protein-stabilised emulsion has attracted research attention, oral tribology in the realm of protein-based emulsions using hydrolysed whey proteins has received limited attention to date (Upadhyay and Chen, 2019). Hydrolysed whey protein, which are known to have a significant effect on improving ageing muscle by providing easily assimilated peptides and essential amino acids, making them particularly appealing for elderly food formulation and specialized diets (Gilmartin, O'Brien and Giblin, 2020; Liu et al., 2015), might also have a role in influencing the lubricity of emulsions when present as an emulsifier in liquid or semi-solid food systems, remaining principally unexplored in the tribological limit. Also, how tribology is altered when whey proteins in combination with their hydrolysed counterparts are used to create model emulsion-filled gels, that represent various food matrices such as cheese and yoghurt, and how mixing with saliva to mimic oral processing remains poorly understood (Fuhrmann et al., 2020a, Fuhrmann et al., 2020b; Shahbazi et al., 2021).

Hence, the aim of this study was to explore the *in vitro* oral tribological characteristics of oil-in-water (o/w) emulsions and emulsion-filled gels containing whey protein isolate and hydrolysed whey protein isolate. We sought to achieve this by modifying the composition and emulsion formation conditions. Specifically, our goal was to develop systems with increased protein content (10 wt% protein), an aspect often overlooked in the literature but crucial for creating geriatric food. We hypothesized that tribological properties could be optimized by modifying the composition to include hydrolysed whey protein isolate instead of native whey protein isolate as emulsifiers. Previous research suggests that hydrolysed whey protein may lead to emulsion droplets that are more prone to shear-induced coalescence in tribological contacts (Dresselhuis et al., 2008; Dresselhuis et al., 2007; Schröder et al., 2017; Singh. and Dalgleish., 1998). Additionally, we employed two colloidal processing routes for creating the o/w emulsions and emulsion-filled gels. One route involved emulsion formation using 10 wt% protein content (M1), while the other began with emulsion formation using 0.1 wt% protein content, followed by protein enrichment to achieve a final concentration of 10 wt% (M2), thus ensuring consistent protein levels. Our hypothesis was that the latter processing route (M2) might render the droplets more susceptible to tribo-shear induced coalescence, thereby enhancing lubricity of a whey protein-rich food matrix. Furthermore, we investigated the impact of model saliva addition on rheological and tribological properties of the emulsions and emulsion gels. We conducted a comprehensive characterization utilizing techniques such as rheology, confocal microscopy, light scattering, to elucidate the underlying mechanisms governing the observed tribological behavior. To the best of our knowledge, this study represents the first attempt to enhance the tribological performance of protein-rich emulsion-based food matrices through compositional and colloidal processing modifications, with potential implications for designing protein-rich foods tailored to vulnerable populations with optimized mouthfeel performance.

## 2 Materials and methods

### 2.1 Materials

Whey protein isolate (WPI) and hydrolysed whey protein (HWP) with 25% degree of hydrolysis (DH 25) containing ≥90% and 85% protein content were obtained from Fonterra Co-operative Group Limited (Auckland, New Zealand) and Power Supplements B.V (Netherlands), respectively. Sodium hydroxide, sodium phosphate monobasic monohydrate, sodium phosphate dibasic anhydrous and hydrogen chloride were purchased from Thermo Fisher Scientific (Loughborough, UK). The lipid phase consisted of sunflower oil (SFO) (Tesco Stores Ltd., UK). 4-(2-hydroxyethyl)-1-piperazineethanesulfonic acid (HEPES) buffer and NaCl were purchased from PanReac AppliChem (Germany) and Fisher Chemicals (UK), respectively. All reagents were of analytical grade and used without further purification unless otherwise reported. The HEPES buffer was prepared with Milli-Q water (Milli-Q apparatus, Millipore, Bedford, UK) with a resistivity of 18.2 MΩ cm at 25 °C. Sodium azide (NaN_3_) (0.02 wt %) was added as a preservative.

### 2.2 Preparation of emulsion, emulsion gels and model boli

#### 2.2.1 Emulsions

Oil-in-water emulsions were prepared using two colloidal processing routes. For M1, powdered WPI, HWP, or a 1:1 combination of WPI and HWP w/w ratio were dispersed and stirred in 20 mM HEPES buffer at pH 7.0 achieving a final protein concentration of 10.0 wt%, at room temperature for 2 h. O/W emulsions were prepared by mixing sunflower oil (SFO) (1–20.0 wt%) with the emulsifier solutions (10.0 wt% in the final emulsions) and subjecting the mixture to pre-homogenization with an Ultra-Turrax T25 (IKA-Werke GmbH & Co., Staufen Germany) rotor-stator system for 1 min at 13,500 rpm. Immediately after pre-homogenization, the pre-emulsions were passed through a high-pressure Leeds Jet homogenizer (University of Leeds, UK) twice at 300 bars to produce the emulsions.

The second preparation process (M2) involved initial emulsion formation with 0.1 wt% protein content, followed by subsequent protein enrichment (9.9 wt%) to reach a final concentration of 10 wt%, ensuring consistent protein levels. In other words, EF0.1 contained WPI, HWP or a combination of WPI and HWP (1:1 w/w ratio) at a final protein concentration of 0.1 wt%. After homogenization, the fresh emulsions were mixed with the remaining WPI, HWP or a combination of WPI and HWP to achieve a final protein concentration of 10.0 wt% at 100 rpm for at least 2 h at room temperature. The WPI, HWP and dual WPI and HWP-stabilised (WPI/HWP)-stabilised o/w emulsions will be referred with the subscript _1_ and _2_ used to refer to M1 and M2, respectively. The nomenclature and the composition are detailed in Table 1.

**Table 1:** Summary of the compositions of the different systems used in this study. Subscripts (_*1 or 2*_) refer to the methods used to produce the samples. Method 1 (_1_) involves creating emulsions with an initial protein content of 10 wt%. In contrast, Method 2 (_2_) starts with an initial protein content of 0.1 wt% for emulsion formation during the homogenization step, which is then enriched to reach a final concentration of 10 wt%.

Sample code	Content (wt%)				
	System Type	Sunflower oil (SFO)	Whey protein isolate (WPI)	Hydrolysed whey protein (HWP)	Model Saliva mixture ratio
WPI_1 or 2_–1 wt% oil	Emulsion	1	10	0	
WPI_1 or 2_–10 wt% oil	Emulsion	10	10	0	
WPI_1 or 2_–20 wt% oil	Emulsion	20	10	0	
WPI_1 or 2_	Emulsion	20	10	0	
WPI_1 or 2 boli_	Emulsion	20	10	0	1:1
WPI_1 or 2 gb_	Emulsion-filled gel	20	10	0	1:1
HWP_1 or 2_–1 wt% oil	Emulsion	1	0	10	
HWP_1 or 2_–10 wt% oil	Emulsion	10	0	10	
HWP_1 or 2_–20 wt% oil	Emulsion	20	0	10	
HWP_1 or 2_	Emulsion	20	0	10	
HWP_1 or 2 boli_	Emulsion	20	0	10	1:1
HWP_1 or 2 gb_	Emulsion-filled gel	20	0	10	1:1
WPI/HWP_1 or 2_	Emulsion	20	5	5	
WPI/HWP_1 or 2 boli_	Emulsion	20	5	5	1:1
WPI/HWP_1 or 2 gb_	Emulsion-filled gel	20	5	5	1:1

The droplet size distribution of the emulsion droplets was determined using static light scattering at 25 °C using a Malvern MasterSizer 3000 (Malvern Instruments Ltd., Malvern, Worcestershire, UK). The refractive index of the sunflower oil (SFO) and the dispersion medium were set at 1.469 and 1.33, respectively. The absorbance value of the emulsion droplets was 0.001. The volume weighted mean average d_43_ (De Brouckere mean diameter) was reported for the oil droplet size.

#### 2.2.2 Emulsion-filled gels

To prepare the emulsion-filled gels, the o/w emulsions stabilised by whey protein isolate (WPI) or hydrolysed whey protein (HWP) or a combination of whey protein isolate and hydrolysed whey protein (WPI/HWP) were incubated in a water bath at 90 °C for 30 min. Samples were then cooled at 25 °C and stored overnight at 4 °C for further analyses. The experimental conditions and composition used are shown in Table 1.

#### 2.2.3 Simulated oral processing

##### 2.2.3.1 Preparation of model saliva

The model saliva (MS) was prepared following the composition previously described by (Sarkar et al., 2009). Briefly, to prepare 1 L of model saliva, 1.59 g L^−1^ NaCl (sodium chloride), 0.328 g L^−1^ NH_4_NO_3_ (ammonium nitrate), 0.64 g L^−1^ KH_2_PO_4_ (potassium dihydrogen phosphate), 0.20 g L^−1^ KCl (potassium chloride), 0.31 g L^−1^ K_3_C_6_H_5_O_7_.H_2_O (potassium citate monohydrate), 0.02 g L^−1^ C_5_H_3_N_4_O_3_Na (uric acid sodium salt), 0.20 g L^−1^ H_2_NCONH_2_ (urea), 0.15 g L^−1^ C_3_H_5_O_3_Na (sodium lactate) and 3.00 g L^−1^ porcine gastric mucin type II were dissolved in distilled water. After adjusting the pH to 7.0 using 1 M NaOH, the volume was made up to 1 L using a volumetric flask. Porcine gastric mucin was used due to the ability of this mucin to simulate the rheological properties of human saliva. Although bovine submaxillary mucin is the optimal source of commercially available mucin for lubricating properties (Sarkar, Xu, & Lee, 2019), this was not used due to cost associated with this purified mucin and therefore this is a limitation of the current study. In addition, α-amylase was not included in the model saliva formulation as starch was not used in any of the formulation of the emulsions and the role of α-amylase was considered to be negligible as seen in previous literature dealing with non-starch polysaccharides (Torres et al., 2018).

##### 2.2.3.2 Model boli preparation

To simulate *in vitro* oral processing of the emulsions, samples were mixed with model saliva at a ratio of 1:1 (w/w) based on previous literature (Agorastos et al., 2022). For the emulsion-filled gel samples, the same 1:1 (w/w) ratio with model saliva was used. These samples were then sheared using a Silverson laboratory mixer L5M-A (Chesham, UK) for 2 min at 2000 rpm at room temperature. Emulsions and emulsion-filled gel boli fragments (see Table 1) were characterized for their droplet size, rheological and tribological properties.

#### 2.2.4 Characterization of emulsions and emulsion-filled gels

##### 2.2.4.1 Rheological measurements

Apparent viscosity (*η*) of the O/W emulsions were conducted using a controlled-stress rheometer MCR 302 (Anton Paar, Austria) equipped with a 50 mm diameter parallel plate geometry (Stokes et al., 2013). The gap was fixed at 1.0 mm and the experimental temperature was kept at 37 °C to mimic the oral conditions. The samples were sealed off with a thin layer of silicone oil to prevent evaporation. Flow curves were obtained between the shear rates (γ) of 0.1–1000 s^−1^. In order to determine the flow type the emulsions, the flow curves were fitted using power-law model (Ostwald-de Waele model).

##### 2.2.4.2 Tribological measurements

The lubrication properties of the samples, emulsions, emulsion boli, and emulsion-filled gel boli, were measured at 37 °C using a Mini-Traction Machine (MTM2, PCS Instruments, UK). The testing set-up consisted of a 1 ball-(19.0 mm diameter)-on-disc contact, with both surfaces made of polydimethylsiloxane (PDMS) with a Young's modulus of 2.4 MPa (Hertzian contact pressure of 343 kPa) (Xu et al., 2020) and average surface roughness of R_a_ ∼50 nm. Prior to the experiments, the PDMS surfaces were cleaned in an ultrasonic bath with a solution of 3% surface-active cleaning agent (Decon 90®, East Sussex, UK), a solution of 10% isopropanol, followed by rinsing with isopropanol. After such treatment, the surface of the PDMS retained its natural hydrophobic characteristic. The entrainment speed (*Ū*) was calculated as $Ū=\frac{U_{B}+U_{D}}{2}$, where *U*_*B*_ and *U*_*D*_ are the ball and disc speeds at the contact point, respectively. Speeds were varied from 1000–0.1 mm s^−1^ (Stokes et al., 2013). Friction coefficients (μ) measurements as a function of entrainment speed (*Ū*) were obtained as the average of three measurements.

##### 2.2.4.4 Confocal laser scanning microscopy (CLSM)

Microstructural observations of emulsions stabilised by whey protein isolate (WPI) or hydrolysed whey protein (HWP) prepared using the two processing approaches M1 (_1_) and M2 (_2_) were made using a Zeiss LSM 880 inverted confocal microscope (Carl Zeiss MicroImaging GmbH, Jena, Germany) using an oil immersion 63 × lens and the pinhole diameter maintained at 1 Airy Unit to filter out the majority of the scattered light. A stock solution of Nile Red (1 mg/mL in dimethyl sulfoxide) was used to stain the oil to a final concentration of 0.02 mg/mL and a stock solution of Fast Green (1 mg/mL in Milli-Q water) was used to stain the protein particles to a final concentration of 0.1 mg/mL. Nile Red and Fast Green were excited at wavelengths of 488 and 633 nm, respectively. The emission filters were set at 555–620 nm for Nile Red and at 660–710 nm for Fast Green. Samples were placed on a concave confocal microscope slide and secured with a glass coverslip before imaging.

#### 2.2.5 Statistics analysis

All measurements were repeated at least three times for two independent samples and data are reported as means ± standard deviation (means ± SD). The statistical analyses were conducted using one-way ANOVA and multiple comparison test using SPSS software (IBM, SPSS statistics, version 24) and the significant difference between samples were considered when *p < 0.05*.

## 3 Results and discussion

### 3.1 Lubrication performance of O/W emulsions affected by oil volume fraction

**Fig. 1:**
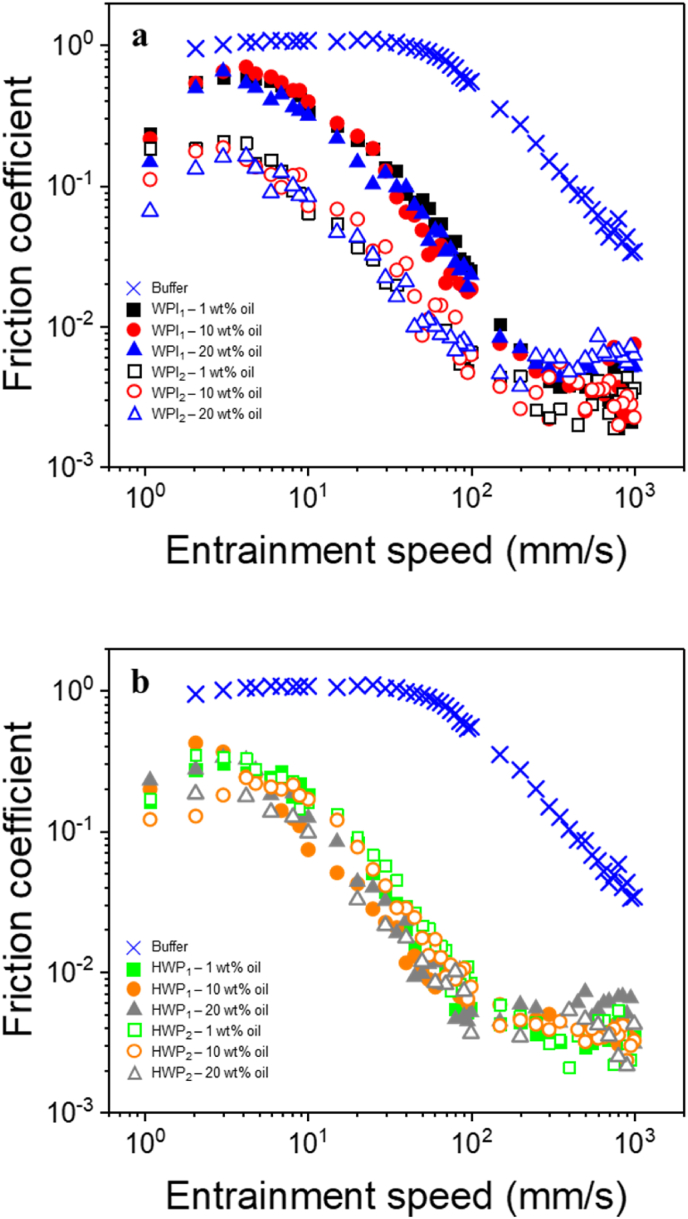
Effect of oil concentration on friction behaviour of o/w emulsions stabilised by (a) whey protein isolate (WPI) and (b) hydrolysed whey protein (HWP) prepared using the two processing routes *i.e.* using 10 wt% protein content initially to form the emulsion (_1_) (filled symbols), and using 0.1 wt% protein content, followed by protein enrichment to achieve a final concentration of 10 wt% (_2_), (open symbols). Friction graphs of HEPES buffer are shown as reference. Data represent mean of triplicate measurements on duplicate samples (n = 2 × 3).

Previous studies have shown that *μ* of o/w emulsions tend to decrease with increasing oil concentration (Chojnicka et al., 2009). However, such emulsions contain low concentrations of protein (Brown et al., 2021). Therefore, we first examined the impact of oil concentration on the morphology and lubrication performance of o/w emulsions that were stabilised using protein types *i.e.* WPI or HWP formed using the aforementioned colloidal processing routes: M1 (_1_) and M2 (_2_) (Fig. 1 and Table 2). In the case of WPI-stabilised of o/w emulsions (WPI), it can be observed that as the oil content was increased from 1.0 wt% to 20.0 wt%, the emulsions consistently exhibited comparable oil droplet sizes (*p* > 0.05). This trend was also apparent for both emulsion formation processing routes (_1_) and (_2_), as observed in Table 2. This result suggests that the oil content did not exert a significant influence on the droplet size, regardless of the emulsion processing used. A similar effect was observed when using HWP as an emulsifier to make the o/w emulsions, as an increase in oil concentration from 1.0 wt% to 20.0 wt% did not affect the oil droplet size under the same processing conditions (*p > 0.05*) (Table 2). However, it is noteworthy that despite the lack of size increase with higher oil concentrations under the same processing conditions, an increase in droplet size was evident between (_1_) and (_2_) methods (*p > 0.05*) (Table 2).

**Table 2:** Effect of oil concentration on mean droplet size *d*_43_ (μm) of O/W emulsions stabilised by different proteins in emulsifier-rich (_1_) and emulsifier-poor (_2_) regimes as fabrication conditions. Different lower case letters in the same column indicate a statistically significant difference (*p < 0.05*).

Protein type	Oil concentration (wt%)	Method 1 (_1_)	Method 2 (_2_)
WPI	1.0	1.30 ± 0.70^a^	1.01 ± 0.14^a^
WPI	10.0	0.81 ± 0.00^a^	1.05 ± 0.01^a^
WPI	20.0	0.711 ± 0.00^a^	2.23 ± 0.03^a^
HWP	1.0	1.12 ± 0.03^a^	9.81 ± 6.28^b^
HWP	10.0	1.71 ± 0.04^a^	14.6 ± 1.39^b^
HWP	20.0	5.65 ± 1.88^a^	16.35 ± 0.24^b^

**Fig. 2:**
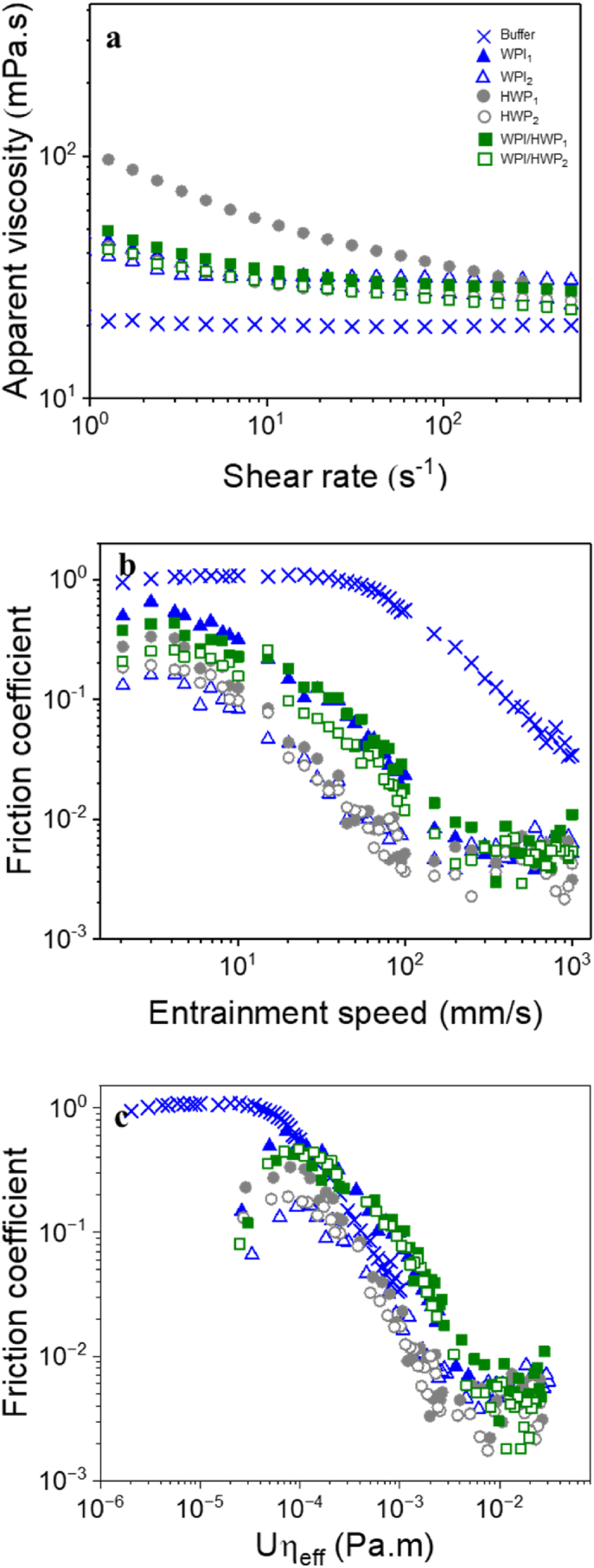
Mean apparent viscosity (ƞ) as a function of shear rate (γ) (a), friction curves (b), friction coefficient as a function of product of entrainment speed × effective viscosity (*Uƞ*_eff_) (c) of o/w emulsions (20.0 wt% oil, 10.0 wt% protein) stabilised by whey protein isolate (WPI), hydrolysed whey protein (HWP) or a mixture of whey protein isolate and hydrolysed whey protein (WPI/HWP) using 10 wt% protein content initially to form the emulsion (_1_) (filled symbols), and using 0.1 wt% protein content, followed by protein enrichment to achieve a final concentration of 10 wt% (_2_), (open symbols). Emulsion formulation and sample preparation method details are listed in Table 1. Data represent mean of triplicate measurements on three samples (n = 2 × 3). Statistics is shown in Table 3.

The impact of oil concentration and the effect of colloidal processing routes, were evaluated based on their lubrication performance (Fig. 1a and b and Fig. 2). In Fig. 1a, the emulsions created with WPI illustrate that, under the (_1_) processing route, an increase in oil concentration did not result in any marked differences (*p* > 0.05) in lubrication behaviour across the entire range of measured speeds. Under (_2_), lubrication behaviour was enhanced compared to the (_1_) method (*p < 0.05*); however, oil concentration showed no discernible impact on lubrication behaviour (*p > 0.05*) (Fig. 1a). In the case of emulsions formulated with HWP, no variations in lubrication behaviour were detected between samples across the entire speed range (*p > 0.05*), regardless of the oil concentration or processing methods used (Fig. 1b). This lack of variation in lubrication behaviour as a function of the oil droplet volume fraction for both the emulsifiers (WPI and HWP) may be explained by the similarity in droplet size when employing the same processing route (Fig. 1 and Table 2). A similar observation was obtained by Wang et al. (2021), where authors observed that oil-in-water emulsions with droplet distributions between 0.01 μm and 10 μm did not show a significantly lower friction coefficient with increasing fat mass fraction in both the boundary and mixed regimes (p > 0.5). The authors speculated that once sufficient droplets are present for entrainment between the two surfaces, further increases in the number of fat droplets provide no additional benefit in surface lubrication. Consequently, from this point forward, we have excluded the influence of oil content on the emulsions, and we opted for a 20.0 wt% oil concentration as a representative droplet volume fraction, which aligns with what is typically found in semi-solid products, such as cheese.

### 3.2 Lubrication performance of O/W emulsions affected by colloidal processing route or protein type

Oil droplet size, flow and tribological properties of 20 wt% oil concentration o/w emulsions are summarised in Table 3, Table 4. Fig. 2a shows the rheological behaviour of the WPI-stabilised, HWP-stabilised and WPI/HWP-stabilised o/w emulsions using the two different emulsion preparation methods (_1_) and (_2_) (see Table 2 for emulsion preparation methods). The Ostwald de Waele model was applied to fit the flow curves and the corresponding fit parameters (consistency coefficient (K), flow index (n), regression coefficient (R^2^) are shown in Table 3. The R^2^ of all samples was ≥0.98, confirming a good fit to the model. All emulsions were pseudoplastic fluid showing shear-thinning behaviour regardless of the colloidal processing route, as indicated by their similar flow index (*n* = 0.82–1). For HWP_1_, it showed the highest *K* and the lowest *n* (Table 3), which suggests strong inter-droplet flocculation. All samples presented nearly the same viscosity at orally relevant shear rate of 50 s^−1^ (*η*_*50*_) (Table 3). We attribute these similarities in the steady shear behaviour to the fixed protein (10.0 wt%) and the oil (20.0 wt%) concentrations that were used to create the emulsion systems, and similar droplet size (*p* > 0.05).

**Table 3:** Mean droplet size and flow properties of o/w emulsions containing 20.0 wt% oil. Different lower case letters in the same column indicate a statistically significant difference (*p < 0.05*).

			Oswald-de Waele fit for the apparent viscosity (*η*_a_)		
			*η*_a_(***γ***) = *K*(***γ***)^n−1^		
Sample	*d*_43_ (μm)	*η*_50_ (mPa.s)	*K*(Pa.s^n^)	*n*	*R*^2^
Buffer	NA	19.75 ± 3.75	0.01 ± 0.00^a^	1 ± 0.00	0.99
SFO	NA	34.30 ± 0.4	0.03 ± 0.00^a^	1 ± 0.00	1
WPI_1_	0.711 ± 0.00^a^	25.67 ± 3.2^a^	0.03 ± 0.00^a^	0.93 ± 0.00	0.99
WPI_2_	2.23 ± 0.03^a^	27.82 ± 2.72^ab^	0.04 ± 0.00^a^	0.96 ± 0.00	1
HWP_1_	5.56 ± 0.24^a^	37.46 ± 1.49^b^	0.08 ± 0.00^b^	0.82 ± 0.00	0.99
HWP_2_	16.35 ± 0.24^b^	26.10 ± 9.97^ab^	0.02 ± 0.00^a^	0.92 ± 0.00	0.99
WPI/HWP_1_	1.90 ± 1.39^a^	30.05 ± 6.78^ab^	0.03 ± 0.00^a^	0.96 ± 0.00	1
WPI/HWP_2_	4.14 ± 1.85^a^	26.57 ± 8.00^ab^	0.03 ± 0.00^a^	0.94 ± 0.00	0.99

**Table 4:** Mean frictional coefficients in presence of emulsion and emulsion gels at entrainment speeds 3, 50 and 100 mm s^−1^ representing boundary and mixed lubrication regimes. Different lower case letters in the same column indicate a statistically significant difference (*p < 0.05*).

Samples	Friction coefficient (*μ*)		
	*μ*_3mm/s_	*μ*_50__mm/s_	*μ*_*10*0__mm/s_
***Reference***			
Buffer	1.0 ± 0.05^d^	0.99 ± 0.02^e^	0.64 ± 0.08^d^
SFO	0.02 ± 0.0^a^	0.003 ± 0.00^a^	0.003 ± 0.00^a^
MS	0.38 ± 0.10^c^	0.20 ± 0.07^bc^	0.05 ± 0.02^a^
***Emulsions***			
WPI_1_	0.48 ± 0.15^cd^	0.06 ± 0.00^a^	0.031 ± 0.03^a^
WPI_2_	0.14 ± 0.09^b^	0.11 ± 0.00^a^	0.003 ± 0.00^a^
HWP_1_	0.03 ± 0.00^ab^	0.06 ± 0.00^a^	0.005 ± 0.01^a^
HWP_2_	0.09 ± 0.07^ab^	0.12 ± 0.16^ab^	0.003 ± 0.00^a^
WPI/HWP_1_	0.29 ± 0.04^ab^	0.32 ± 0.02^c^	0.01 ± 0.01^a^
WPI/HWP_2_	0.47 ± 0.04^c^	0.07 ± 0.00^a^	0.009 ± 0.02^a^
***Emulsion boli***			
WPI_1 boli_	0.02 ± 0.10^a^	0.07 ± 0.00^ab^	0.04 ± 0.02^a^
WPI_2 boli_	0.25 ± 0.17^a^	0.04 ± 0.01^a^	0.17 ± 0.30^a^
HWP_1 boli_	0.42 ± 0.24^a^	0.02 ± 0.00^a^	0.007 ± 0.01^a^
HWP_2 boli_	0.28 ± 0.08^a^	0.03 ± 0.01^a^	0.008 ± 0.04^a^
WPI/HWP_1 boli_	0.39 ± 0.03^b^	0.03 ± 0.01^a^	0.22 ± 0.17^bc^
WPI/HWP_2 boli_	0.56 ± 0.15^b^	0.41 ± 0.10^d^	0.07 ± 0.03^a^
***Emulsion-filled gel boli***			
WPI_1 gb_	0.68 ± 0.02^cd^	0.38 ± 0.04^cd^	0.30 ± 0.03^c^
WPI_2 gb_	0.87 ± 0.00^d^	0.10 ± 0.03^ab^	0.10 ± 0.10^b^
WPI/HWP_1 gb_	0.04 ± 0.04^ab^	0.16 ± 0.05^ab^	0.13 ± 0.01^b^
WPI/HWP_2__gb_	0.06 ± 0.02^ab^	0.02 ± 0.00^a^	0.02 ± 0.03^a^

Abbreviations: SFO = sunflower oil; MS = model saliva; WPI = whey protein isolate; HWP = hydrolysed whey protein.

**Fig. 3:**
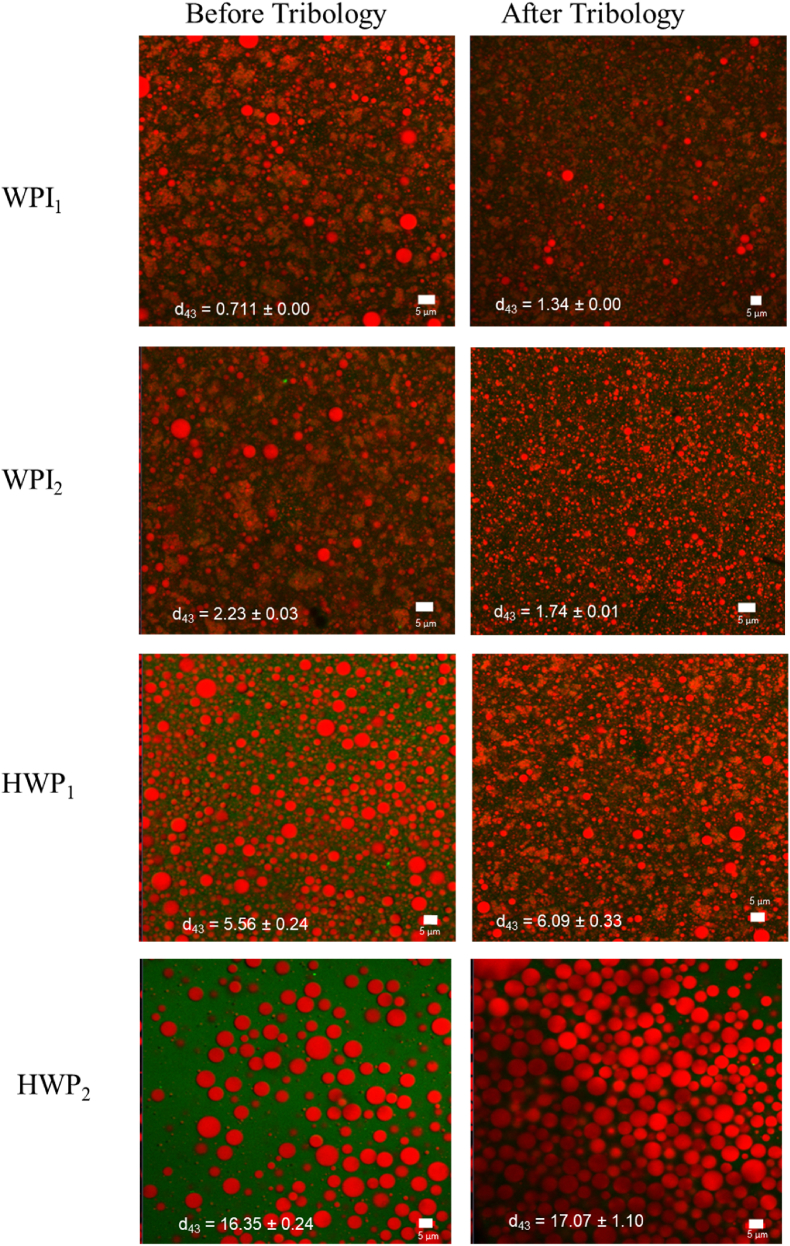
Confocal laser scanning microscopy (CLSM) images with insets of mean droplet size (*d*_43_, μm) of emulsions (20.0 wt% oil) stabilised by 10.0 wt% whey protein isolate (WPI) or hydrolysed whey protein (HWP) made using 10% protein content initially to form the emulsion (_1_), and using 0.1 wt% protein content, followed by protein enrichment to achieve a final concentration of 10 wt% (_2_). Images are taken before and after tribological measurements. Red channel shows the Nile Red signal coming from the oil droplets and green channel shows the Fast Green signal coming from the proteins. (For interpretation of the references to colour in this figure legend, the reader is referred to the Web version of this article.)

Fig. 2b shows the tribological behaviour of the WPI-stabilised, HWP-stabilised and WPI/HWP-stabilised o/w emulsions using the two different colloidal processing routes. For WPI formed using the two different emulsion preparation methods, WPI_1_ and WPI_2_, both systems showed a boundary regime at ≤ 3 mm s^−1^ and a decreased friction in the mixed regime at 3 ≤ *Ū* ≤ 550 mm s^−1^. For WPI_2,_ *μ* in the boundary region (*Ū* = 3 mm s^−1^) was lower compared to WPI_1_ (*p* < 0.05) (0.48 ± 0.15 and 0.14 ± 0.09 for WPI_1_ and WPI_2_, respectively) (Fig. 2b, Table 4). This effect was not obvious at *Ū* = 50 mm s^−1^ nor 100 mm s^−1^, when both WPI_1_ and WPI_2_ were found to be in the mixed lubrication regime, with *μ* for WPI_1_ being similar (*p* > 0.05) with respect to the *μ* values for WPI_2_ (Fig. 2b, Table 4). Differences on the lubrication behaviour between the colloidal processing routes *i.e.* (_1_) and (_2_) used might be attributed to surface-induced coalescence (Fujita and Kimura, 2013). Previous studies on the influence of bulk proteins on the lubrication properties of o/w emulsions stabilised using whey protein in presence of hydrophobic PDMS surfaces, have shown that unstable emulsions lubricate the surface better than the stable emulsion, independent of the bulk protein concentration (Dresselhuis et al., 2007). In the study, it was reported that the contrast in friction observed between shearing a 0.3-wt% stabilised emulsion and a 1-wt% stabilised emulsion arises from the predominant effect of oil spreading triggered by surface-induced coalescence, irrespective of the bulk protein concentration (0–1 wt%). In our study, oil droplets did not appear to coalesce during the tribology measurements, as no free oil could be observed visually or under the CLSM (Fig. 3). However, studies have shown that only a small amount of oil is needed to form a lubricating layer, so it is possible that o/w emulsions formed using (_2_) processing route (WPI_2_) might have formed few unstable droplets due to partial protein coverage resulting from the reduced protein concentration used in their formation, despite protein enrichment. Thus, these oil droplets might have undergone some degree of coalescence improving the lubrication as compared to WPI_1_ (Dresselhuis et al., 2007; Liu and Tan, 2012).

For HWP systems, entrainment speed *Ū* = 3 mm s^−1^ corresponded to the boundary regime, and *μ* was 0.03 ± 0.00 and 0.09 ± 0.07 for HWP_1_ and HWP_2,_ respectively (*p* > 0.05) (Fig. 2b). At both *Ū* = 50 mm s^−1^ and 100 mm s^−1^, corresponding to the mixed lubrication regime, *μ* was also similar for HWP_1_ and HWP_2_ (0.06 ± 0.00 and 0.12 ± 0.01, respectively at 50 mm s^−1^ and 0.005 ± 0.01 and 0.003 ± 0.0, respectively at 100 mm s^−1^) (*p* > 0.05) (Table 4). Hence, for HWP, colloidal processing route did not seem to influence the frictional behavior in both, boundary, and mixed lubrication regimes. Hydrolysis of whey proteins is known to change the surface hydrophobicity of proteins and reduce their stabilising effect at the oil-water interface. Euston et al. (2001) found that whey protein hydrolysates with low or high degree of hydrolysis (DH) (4–10% or 27–35%) have poorer emulsifying ability compared to native whey protein, whereas whey protein hydrolysates with an intermediate DH (20–27%) showed improved emulsion forming ability. In addition, Singh. and Dalgleish. (1998), reported that the maximum emulsifying capacity was obtained from whey protein hydrolysates with a 10 or 20% DH, and that higher DH resulted in peptides that were too short to act as effective emulsifiers. Since hydrolysis produces a range of peptides, it is possible that the peptides from the whey protein hydrolysates used in this study (DH 25% as reported by the manufacturer) had poor emulsifying ability. This could potentially account for the observed increase in droplet size, which was rather obvious even before subjecting to tribological stress (Fig. 3). Hence, it is reasonable to assume that, irrespective of their colloidal processing, HWP-stabilised emulsions were, to some extent, more sensitive to coalescence due to their increased size, and therefore, showed improved lubrication properties compared to WPI-stabilised emulsions (Fig. 1, Fig. 2b).

It was also intriguing to combine WPI and HWP to understand whether that might have an influence on lubrication performance. Upon combination of WPI and HWP to form o/w emulsion (WPI/HWP), reduced *μ* in the boundary lubrication regime (*Ū* = 3 mm s^−1^) was observed for WPI/HWP_1_ as compared to WPI/HWP_2_ (0.29 ± 0.04 and 0.47 ± 0.04 for WPI/HWP_1_ and WPI/HWP_2_, respectively (*p* < 0.05) (Fig. 2b and Table 4). In addition, in the mixed regime at *Ū* = 50 mm s^−1^, for WPI/HWP_2_ lubrication was decreased compared to WPI/HWP_1_ (0.32 ± 0.02 and 0.07 ± 0.0 for WPI/HWP_1_ and WPI/HWP_2_, respectively (*p* < 0.05), with a minimal effect on WPI/HWP_2_ at high speeds (*Ū* = 100 mm s^−1^) (0.01 ± 0.01 and 0.009 ± 0.02 for WPI/HWP_1_ and WPI/HWP_2_, respectively (*p* > 0.05) (Fig. 2b and Table 4). Comparing the friction coefficient (*μ*) of the mixtures to the single systems made with (_1_) processing route (WPI_1_ and HWP_1_), we can observe that the measured friction curves of the WPI/HWP_1_ overlapped with the WPI_1_ curve, indicating that for WPI/HWP_1_, the lubricating behaviour was dominated by the properties of the WPI, such as protein interaction and structural properties, rather than the properties of HWP (Fig. 2b). For samples made with (_2_), the friction curve of WPI/HWP_2_ was higher than those of WPI_2_ and HWP_2_ in single systems, indicating that lubrication properties of the o/w emulsions were negatively affected by the combination of WPI and HWP molecules (1:1 w/w ratio) (Fig. 2b).

Since transition between the boundary and mixed lubrication regimes is known to be influenced by the effective viscosity of the lubricating fluid, we scaled the data of Fig. 2b to viscosity in Fig. 2c showing *μ versus* the product of viscosity and entrainment speed (effective viscosity, *Uη*_*eff*_) using the shear rates (1000 s^−1^) used by Andablo-Reyes et al. (2019). This approach was employed based on their findings, which demonstrated that, for complex fluids exhibiting shear rate-dependent viscosity, the high shear rate limit viscosity (1000 s⁻^1^) serves as a suitable approximation for quantifying the viscous forces of complex fluids in the tribological contact. From this plot, we see that the Stribeck curves for the WPI-stabilised, HWP-stabilised and WPI/HWP-stabilised emulsions reasonable overlap in the mixed lubrication regime regardless of the processing method (Fig. 2c). This overlapping trends in the Stribeck analysis in the mixed regime confirms the role of viscosity in the lubrication phenomena, in agreement with the similar flow behaviour properties observed (*η*_*50*_) (*p* > 0.05) (Table 3).

To recap, the impact of emulsion processing method on lubrication properties was more pronounced for whey protein isolate emulsions (WPI), with (_2_) showing improved lubrication behaviour as compared to (_1_) method. Enhanced lubrication in the (_2_) might be attributed to the presence of free oil. For emulsion made with hydrolysed whey protein (HWP_1_ and HWP_2_), processing methods had no significant effect on the lubrication behaviour. However, enhanced lubrication properties of both systems can be attributed to the reduced physical stabilising effect of HWP at the oil-water interface When replacing 50% of WPI with HWP to make an emulsion (WPI/HWP), lubrication properties were negatively impacted for both processing methods as compared to their single system counterpart.

### 3.3 Effect of model saliva on lubrication properties of O/W emulsion boli

**Fig. 4:**
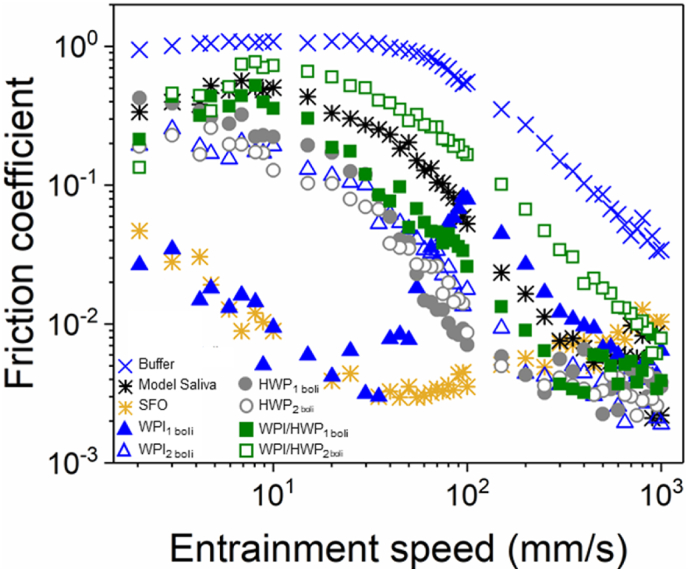
Effect of model saliva (MS) on frictional behaviour of o/w emulsion boli (20.0 wt% oil, 10.0 wt% protein) stabilised by whey protein isolate (WPI), hydrolysed whey protein (HWP) or a mixture of whey protein isolate and hydrolysed whey protein (WPI/HWP). Emulsion formulation and sample preparation method details are listed in Table 1. Data represent mean of triplicate measurements on three samples (n = 2 × 3). Statistics is shown in Table 4.

The inclusion of model saliva (MS) enables the system to have more relevance to oral conditions but also adds additional complexity to understand the mechanism due to multiple interactions taking place. Fig. 4 shows the lubrication performance of the MS used and the effect of MS addition on the *μ* of the o/w emulsion *i.e.* o/w emulsion boli under the different processing methods. MS showed reduced *μ* as compared to those of the buffer throughout the entrainment speeds (Fig. 4, Table 4). This observation implies that the mucin glycoproteins utilized for the MS preparation adsorbed to some extent to the PDMS surfaces. Salivary mucins (MUC5B and MUC7) are recognized for their capacity to adsorb and provide a protective and lubricating layer attributed to their high molecular weight and hydration level, which arises from the existence of regions that are highly glycosylated (Gibbins et al., 2014).

The lubrication properties of the o/w emulsions after model saliva (MS) addition are also shown in Fig. 4. For emulsion boli samples made using the (_1_) route (WPI_1 boli_), a fiction curve different from classic Stribeck curve was observed. At the beginning (*Ū* < 20 mm s^−1^), *μ* was significantly reduced, which is consistent with the mixed regime in the conventional Stribeck curve (Fig. 4 and Table 4). With increasing sliding speed (*Ū* > 20 mm s^−1^), the *μ* increased significantly behaving similarly to the friction curve of the MS. Given the similar behaviour to the SFO curve at low *Ū* (<20 mm s^−1^), it is reasonable to assume that the deviation from the conventional shape of the Stribeck curve at low *Ū* might have been dominated by presence of some free oil. As mentioned before, at low speeds small amounts of oil have been observed to form a lubricating continuous film within the contacting surfaces, caused by droplet coalescence. This has been described in terms of the more favourable wetting properties of the oil over the aqueous phase in hydrophobic surfaces, such as PDMS (de Vicente et al., 2005). This characteristic closely mimics the interaction of oil with the hydrophobic surfaces of the oral cavity, suggesting that the observed lubrication behavior can have direct implications for the sensory perception of food.

Above a critical speed (*Ū* > 20 mm s^−1^), the oil most likely was replaced by a water-continuous phase resulting in a drastic decrease in elastohydrodynamic (EHL) film thickness and a sharp increase in friction. This critical speed has been seen to depend on oil viscosity, surfactant type and oil phase volume, which makes the interpretation of the behaviour of emulsions in lubricating contacts complex (Schmid and Zhou, 2000; Szeri, 1996). Further research is required for an in depth understanding of the response of each phase and component to fully interpret this result. However, similar behaviour in yoghurts and emulsion gel boli have also been reported where only boundary regime behaviour is dominated by the oil phase (Huang et al., 2020; Mu et al., 2022; Nguyen et al., 2017). For WPI_2 boli_, the friction behaviour was between those of WPI_1_ (without MS) and of model saliva (MS) at both boundary and mixed regimes (Fig. 4 and Table 4). As no visual observation of separation or changed in droplet size were observed (data not shown), the lubrication behaviour could be explained by dilution effects (*p* > 0.05) (Torres et al., 2018). The dilution of the emulsion with MS reduces the volume fraction of oil entrained in between the tribo-pairs, as well as the protein absorbed which leads to a higher fluid friction. Furthermore, the *μ* of WPI_2 boli_ was still lower compared to MS in the boundary regime, suggesting that oil droplets entered the gap and separated the two contact surfaces (*p* < 0.05) (Fig. 4 and Table 4). When model saliva was added to HWP to create a boli (HWP_boli_), the emulsions prepared using (_2_) (HWP_2 boli_) (*p* < 0.05) showed lower boundary lubrication reduction versus HWP_1 boli_ (Fig. 4 and Table 4) unlike the systems without model saliva (MS), where no effect between the regimes on the *μ* was observed (Table 4).

Similarly, to the separate lubrication performance of WPI_boli_ and HWP_boli_, the lubrication of the binary emulsion bolus systems (WPI/HWP_boli_) also showed changes of the *μ* for WPI/HWP_1 boli_ in the mixed regime as compared to their emulsion counterpart (without saliva). For WPI/HWP_2 boli_ samples, an increased (*p* < 0.05) in *μ* in the mixed lubrication regime as compared to their emulsion counterpart (without saliva) and their single system equivalents (HWP _boli_) was observed, as shown in Fig. 4 and Table 4. Dresselhuis et al. (2007) also investigated the influence of salivary proteins on the tribological behaviour of o/w emulsions and observed that saliva components, such as the mucin glycoproteins, behave similar as bulk proteins regarding adherence, which resulted in an increase in friction. To sum it up, it is evident that the addition of model saliva has a significant impact on the increasing friction dissipation of emulsions stabilised by either WPI or HWP or WPI/HWP. In particular, differences in friction between colloidal processing route or protein type were affected upon addition of saliva (Table 4).

### 3.4 Lubrication properties of emulsion-filled gel boli

**Fig. 5:**
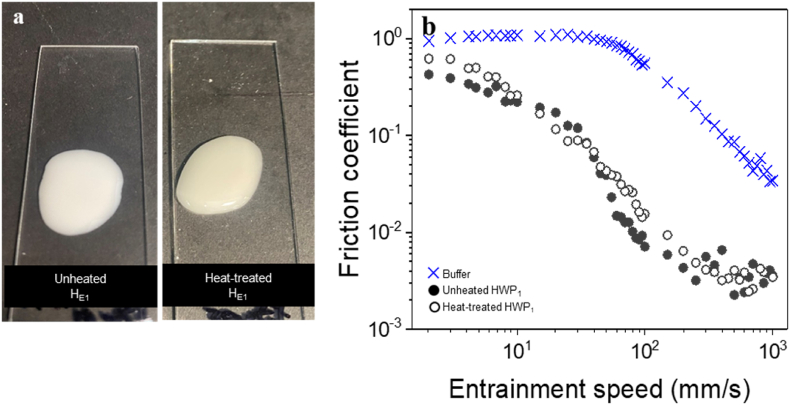
Visual appearance (a) and mean frictional coefficients (b) as a function of entrainment speed of o/w emulsion stabilised by hydrolysed whey protein (HWP) (10.0 wt% protein, 20.0 wt% oil) using 10% protein content initially to form the emulsion (_1_) as fabrication conditions before (unheated) and after heat-treatment (pH 7, 90 °C, 30 min). Data represent mean of triplicate measurements on three samples (n = 2 × 3). Statistics is shown in Table 4.

Initially, we investigated whether the emulsion stabilised by hydrolysed whey protein (HWP) could undergo gelation upon heat treatment. Previous studies have indicated that whey protein hydrolysates may lose their ability to form heat-induced gels; specifically, hydrolysates with a 20% degree of hydrolysis (DH) did not exhibit gelation (Severin and Xia, 2006). Therefore, it was essential to assess how the heat treatment conditions used in our study (pH 7.0, 90 °C for 30 min) would influence the gelling behavior and lubrication properties of both HWP_1_ and HWP_2_. Post heat treatment, both HWP_1_ and HWP_2_ displayed fluid-like behavior, suggesting that the 25% DH, as reported by the manufacturer, significantly affected the gelling ability of the hydrolysed protein (Fig. 5a). Furthermore, the lubrication properties of the heat-treated samples showed no significant difference compared to non-heat-treated samples, as depicted in Fig. 5b. Consequently, emulsion-filled gel systems stabilised by HWP were excluded from further analysis.

**Fig. 6:**
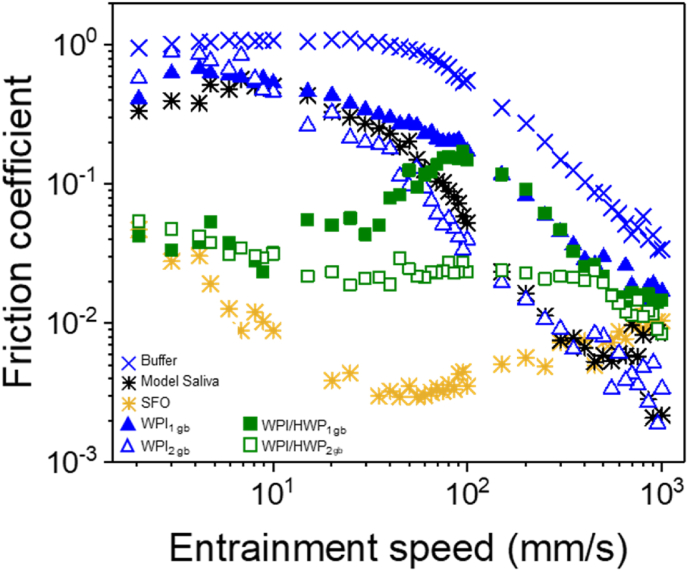
Effect of model saliva (MS) on friction coefficient curves of o/w emulsion-filled gel boli (_gb_) stabilised by whey protein isolate (WPI) or a mixture of whey protein isolate and hydrolysed whey protein (WPI/HWP). Data represent mean of triplicate measurements on duplicate samples (n = 2 × 3). Emulsion formulation and sample preparation method details are listed in Table 1. Statistics is shown in Table 4.

The lubrication behaviour of the emulsion-filled gel boli of WPI and the dual WPI/HWP systems are shown in Fig. 6. Heat-treatment of the WPI and WPI/HWP emulsions to form their corresponding emulsion-filled gel resulted in WPI-stabilised emulsion-filled gels (WPI _gel_) forming a white, opaque and rigid structure, whereas emulsion-filled gels formed from WPI/HWP-stabilised emulsions were more fragile and could be easily broken down (Supplementary Fig. S1). This is perhaps not surprising, as cohesive, and stiff gels are known to be formed by whey protein isolate solutions of high protein concentration (>5 wt%) containing oil droplets, and mixing hydrolysate whey protein with whey protein isolate was expected to render less cohesive gels (Rosa et al., 2006).

Upon mixing with MS to create a bolus (_gb_), irrespective of the formation processing route, WPI _gb_ particles were not homogenous, resulting in a large variation in the size of the gel particles. Both, WPI_1__gb_ and WPI_2 gb_ samples broke down into pieces of different mm sizes and a continuous phase was observed (Supplementary Fig. S1). Hence, the observed tribological behaviour for both boli samples, irrespective of their formation process used can be explained by the fact that large gel particles did not enter the contact zone between the two tribo-pairs (Fig. 6). Hence, the lubrication mechanisms in both cases could be attributed to the dominant effect of the exuded continuous/model saliva phase. This is evidenced by the similarity in the overlap lubrication behaviour between the bolus and the MS across the entire range of speeds, especially for sample made using (_2_) (as depicted in Fig. 6). For sample made using (_2_), it is possible that the relatively hard and non-spherical structure of the gel particles may have increased the surface roughness and friction as seen in Fig. 6. Similar results have previously been reported for emulsion-filled gels with clustered oil droplets during mimicked oral processing (Fuhrmann et al., 2020a, Fuhrmann et al., 2020b).

Interestingly, in contrast to the systems made using WPI, when mixed with HWP to create a dual emulsion-filled gel bolus WPI/HWP _gb_, they presented a significantly different tribological behaviour (*p* < 0.05) (Fig. 6 and Table 4). Differences on the fabrication methods utilized in creating the emulsion-filled gels on the lubrication curves can be observed, particularly at *Ū* > 50 mm s^−1^ where significant different *μ* values at *Ū* = 100 mm s^−1^ can be observed (*p* < 0.05) (Table 4). For WPI/HWP_1 gb_, we can see again that it displayed a friction curve different from classical Stribeck curves. In comparison, WPI/HWP_2 gb_ only displayed a mixed regime throughout the entrainment speed (Fig. 6). As outlined in the section above for the emulsion bolus systems, the lubrication behaviour mechanism for WPI/HWP_1 gb_ can potentially be explained by a speed-dependent *μ* dominated by the presence of visibly weaker gel resulting from the incorporation of hydrolysed whey protein or by the continuous phase. This modification of the gel strength leads to the formation of a gel that is able to deform and flatten out more readily, entering more easily between the tribo-pairs gap separating the ball and disc at a lower entrainment speed and reducing *μ* at lower entrainment speeds, unlike the WPI_1 gb_ that exhibit bigger gel particles (Supplementary Fig. S1). According to this, it is proposed that, as the speed increase, more gel particles were excluded from the sliding interface. Thus, above a critical speed (*Ū* < 200 mm s−1), the gel particles most likely were replaced by the water-continuous phase resulting in a drastic increase in friction, as seen by the overlap on the lubricating behaviour with MS (Fig. 6).

The observation of lower *μ* in samples made using (_2_), compared to (_1_) processing route suggests the action of a distinct lubrication mechanism (Fig. 6 and Table 4). Possibly, in addition to the proposed small gel particles that are able to deform and enter the contact zone more readily, some oil droplets might have released and aided in the reduction of friction at higher entrainment speeds *Ū* < 200 mm s^−1^. A similar significant reduction in *μ* at *Ū* < 100 mm s^−1^ has been observed for starch microgel particles with and without oil after α-amylase addition (Torres et al., 2018).

**Fig. 7:**
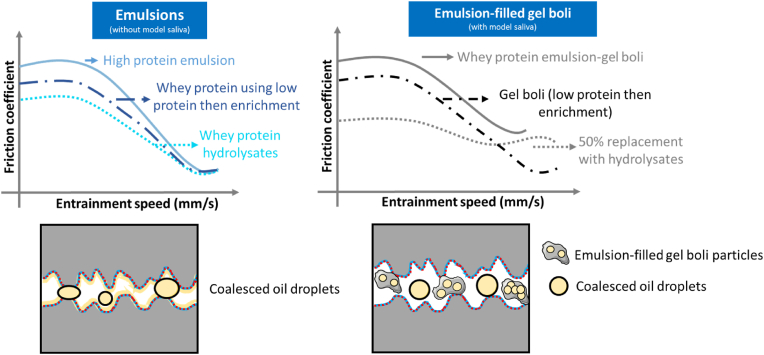
Schematic illustration of importance of using whey protein hydrolysate or fabricating the systems using 0.1 wt% protein content, followed by protein enrichment to achieve a final concentration of 10 wt% in preparation of high protein oil-in-water emulsions and emulsion gels. The top drawing illustrates the friction behavior trend observed during tribological analysis of the emulsions and emulsion-filled gel boli. The bottom diagrams displays the lubrication mechanism for both systems. Blue and red lines depict the adsorption of whey protein isolate (WPI) and hydrolysed whey protein (HWP) films onto the surfaces. For the left drawing, an oil film coats the surface, corresponding to the coalescence observed in emulsions prepared using HWP, which contributes to enhanced lubrication. In contrast, the right drawing depicts the lubrication mechanism for emulsion-filled gel boli. Here, the reduced oil release from the gel particles results in increased friction behaviour. (For interpretation of the references to colour in this figure legend, the reader is referred to the Web version of this article.)

We have now described the findings schematically in Fig. 7. In high protein emulsions, friction can be reduced either by using HWP as the emulsifier or by using WPI in an (_2_) colloidal processing route. The main mechanism might be to induce some degree of droplet coalescence to form a lubricating tribofilm. Even such lubrication performance is restored particularly when mixed with saliva in case of emulsions stabilised by HWP. In case of emulsion gels, preparation *via* (_2_) route helps in reducing friction of emulsion gel boli particularly in the mixed regime. Of more importance, replacing 50% of WPI by HWP emulsion-filled gel boli exhibit a significant reduction in boundary *μ* values.

## 4 Conclusions

This study employed a combination of droplet size, rheology and tribology analysis to elucidate the lubrication mechanisms in high-protein containing oil-in-water emulsions, both in liquid and semi-solid forms. Our findings highlight the significant impact of emulsion processing methods on lubrication properties, with variations observed across different whey protein types and processing methods. In particular, formulating emulsions either using whey protein hydrolysate or whey protein isolate by initially preparing emulsion with 0.1 wt% protein content followed by subsequent protein enrichment can be an effective to destabilize the emulsions in tribological shear and create a coalesced tribofilm reducing friction. Furthermore, the incorporation of model saliva emerged as a crucial factor influencing tribological behaviour, intricately linked to emulsion composition as well as preparation methods. For dual protein emulsion systems, complex adhesion properties appeared to govern lubrication behaviour, warranting in-depth exploration of the underlying mechanism. In emulsion-filled gel boluses, substituting 50% of whey protein with hydrolysed whey protein led to significant alterations in lubrication properties. These findings contribute to our understanding of the importance of protein type and processing when formulating high-protein containing liquid and semi-solid model food systems with optimized mouthfeel, particularly for vulnerable populations. Ongoing studies are looking at the sensory properties of these emulsions and emulsion gels to validate the *in vitro* oral processing findings from this study.

## CRediT authorship contribution statement

**Andrea Araiza-Calahorra:** Conceptualization, Methodology, Investigation, Data curation, Formal analysis, Writing – review & editing, Visualization, Writing – original draft, Writing – review & editing. **Alan R. Mackie:** Writing – review & editing, Supervision. **Anwesha Sarkar:** Conceptualization, Project administration, Writing – review & editing, Supervision, Funding acquisition.

## Declaration of competing interest

No conflicts of interests.

## Data Availability

Data will be made available on request.
