# Supplementary material for: Oral tribology of dairy protein-rich emulsions and emulsion-filled gels affected by colloidal processing and composition
Source: Curr Res Food Sci. 2024 Jul 14;9:100806. doi: 10.1016/j.crfs.2024.100806 (PMC11324994; doi:10.1016/j.crfs.2024.100806)
Supplement: Multimedia component 1 [file mmc1.docx]

**Oral tribology of dairy protein-rich emulsions and emulsion-filled gels affected by colloidal processing and composition**

Supplementary Tables and Figures


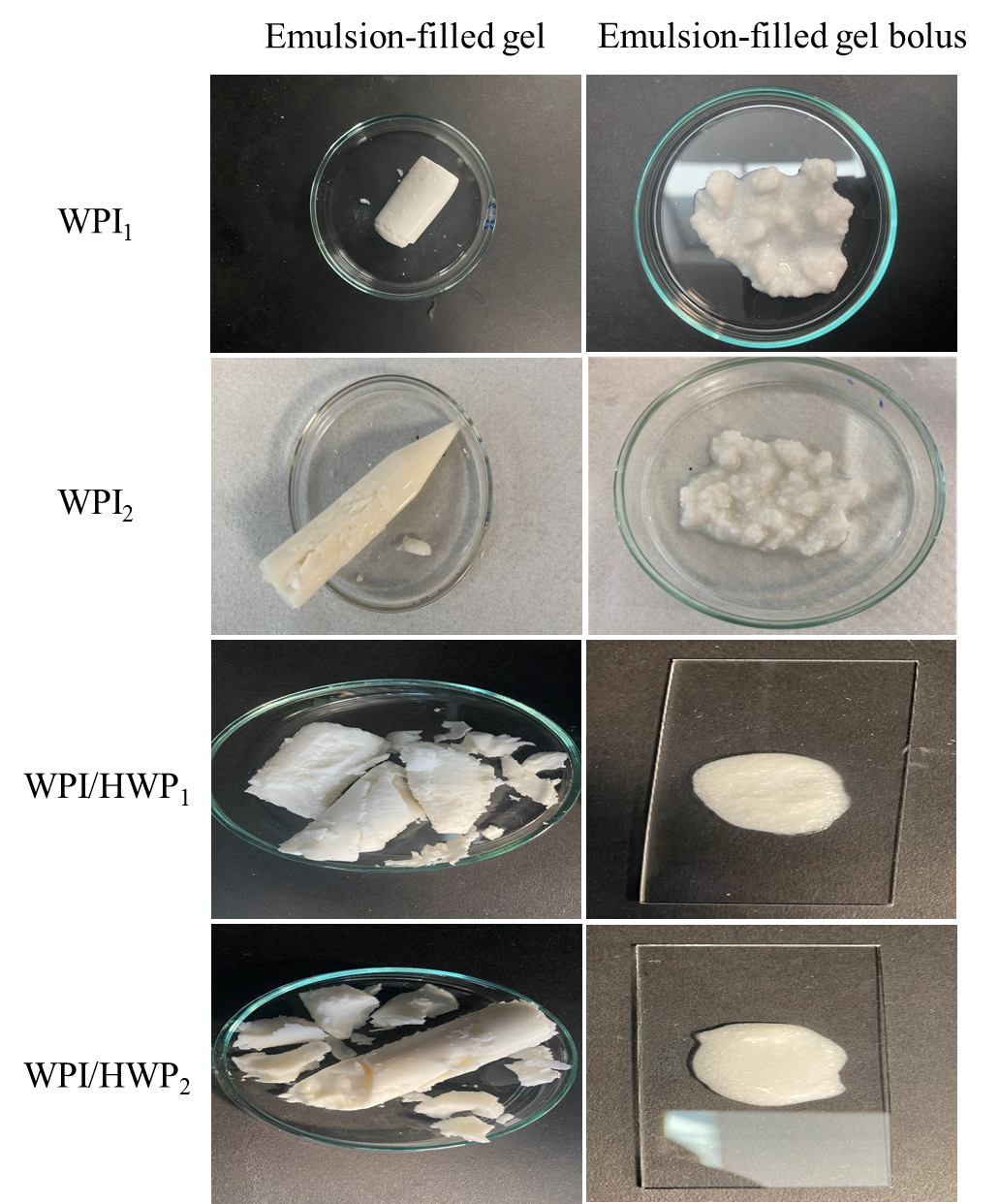


**Fig. S1.** Visual appearance of o/w emulsion-filled gels stabilised by WPI and WPI/HWP mixtures (10.0 wt% protein, 20.0 wt% oil) using 10 wt% protein content initially to form the emulsion (1) (filled symbols), and using 0.1% protein content, followed by protein enrichment to achieve a final concentration of 10 wt% (2), as the two fabrication methods before and after bolus formation (emulsion-filled gels boli (_gb_)).
